# Sexual Violence in the Media: An Exploration of Traditional Print Media Reporting in the United States, 2014–2017

**DOI:** 10.15585/mmwr.mm6947a1

**Published:** 2020-11-27

**Authors:** Olivia Egen, Laura M. Mercer Kollar, Jenny Dills, Kathleen C. Basile, Bethlehem Besrat, Laura Palumbo, Kellie E. Carlyle

**Affiliations:** ^1^Association of Schools and Programs of Public Health, Washington, D.C.; ^2^Division of Violence Prevention, National Center for Injury Prevention and Control, CDC; ^3^Department of Behavioral Sciences and Health Education, Rollins School of Public Health, Emory University, Atlanta, Georgia; ^4^National Sexual Violence Resource Center, Harrisburg, Pennsylvania; ^5^Department of Health Behavior and Policy, School of Medicine, Virginia Commonwealth University, Richmond, Virginia.

Sexual violence is prevalent and, for many victims, begins early in life (*1*). In the United States, one in five women and one in 38 men report completed or attempted rape victimization during their lifetime, with 43.2% of female and 51.3% of male victims reporting that their first rape victimization occurred before age 18 years (*1*). Media have been shown to act as a socializing agent for a range of health and social behaviors (*2*). Media portrayals might influence, reinforce, or modify how the public responds to incidents of sexual violence and their support for prevention efforts and media might construct a lens through which the public can understand who is affected by sexual violence, what forms it takes, why it happens, and who is responsible for addressing it (*3*). Media portrayals of sexual violence were assessed using a systematic random sample of newspaper articles from 48 of the top 50 distributed traditional print media outlets that were examined for sexual violence content and potential differences by geographic region and year of publication. Differences by year and region in type of sexual violence covered, media language used, and outcomes reported were identified, highlighting an opportunity for public health officials, practitioners, and journalists to frame sexual violence as a preventable public health issue and to incorporate best practices from CDC and the National Sexual Violence Resource Center’s Sexual Violence Media Guide (*4*).

Whereas numerous studies describe media portrayals of sexual violence and other forms of violence (*5*–*7*), none examined regional or temporal differences in coverage. This study used 27 sexual violence-related terms* to identify a systematic random sample of 2,600 articles from 48 of the top 50 traditional print media outlets distributed in the United States (*8*) available via electronic newspaper databases.^†^ Outlets were stratified by regional or nationwide reach, and equal systematic samples of 130 articles were selected from each stratum for each publication year, 2014–2017. Articles were coded for strata represented and year published, type of sexual violence mentioned (sexual assault, rape, child sexual abuse, sexual exploitation, sex trafficking, prostitution, sexual harassment, or child pornography), what Sexual Violence Media Guide language was used (sex scandal/scandal, sex/intercourse, accuser, or accused) (*4*), and outcomes. Outcomes included perpetrator consequences (criminal justice system, civil justice system, social, or business consequences) and prevention messaging (primary, secondary, and tertiary prevention). The codebook development relied on the Sexual Violence Media Guide, which provides relevant information for effective communication about sexual violence (*4*). The guide is grounded in media language recommendations from the Maine Coalition Against Sexual Assault (*4*), CDC’s Stop SV: A Technical Package to Prevent Sexual Violence (*9*), and past similar research (*5*,*6*). Media language considerations include suggested language (e.g., “alleged perpetrator” or “perpetrator” if convicted) and language to avoid (e.g., “accused”). Two coders were trained, and intercoder reliability was assessed on 20% of the sample, resulting in an average Kappa = 0.81, and the remaining sample was randomly split between the coders and coded. Analysis of variance (ANOVA) and post-hoc Tukey comparisons were made by article characteristic (region or year) for the type of sexual violence mentioned, media language used/language to avoid, and outcomes. Codes were not mutually exclusive.

The types of sexual violence mentioned in newspaper articles (Table 1) differed significantly by region (Table 2). The percentage of articles within each region covering child sexual abuse was lower nationwide (28.5%) than in the Midwest (38.3%) and Northeast (42.9%) regions. National outlets published a significantly higher percentage of articles on sexual harassment (27.7%) than did media in all other regions (11.5% to 19.2%). National outlets used the term “sex scandal” or “scandal” more frequently than did media in all four regions (11.0% versus 3.5%–6.0%). The percentage of articles using the term “sex” or “intercourse” was higher in national outlets (17.1%) than in media in the Midwest (10.8%), Northeast (8.5%), and West (9.6%) regions. Inclusion of consequences for perpetrators was similar in all regions; however, calls for primary prevention of sexual violence were more frequent in national media articles (12.5%) than in those published in the Northeast (6.0%), South (6.0%), and West (7.3%).

**TABLE 1:** Sexual violence in traditional print media, newspapers, by geographic region — United States, 2014–2017

Region/States*	Newspapers in region*
Nationwide	
National distribution	The Los Angeles Times
	The New York Times
	USA Today
	The Wall Street Journal
	The Washington Post
**Midwest**	
Illinois, Indiana, Iowa, Kansas, Michigan, Minnesota, Missouri, Nebraska, North Dakota, Ohio, South Dakota, Wisconsin	Chicago Sun Times
	Chicago Tribune
	Detroit Free Press
	Milwaukee Journal Sentinel
	St. Louis Post-Dispatch
	Star Tribune
	The Cincinnati Enquirer
	The Columbus Dispatch
	The Indianapolis Star
	The Kansas City Star
	The Plain Dealer
**Northeast**	
Connecticut, Maine, Massachusetts, New Hampshire, New Jersey, New York, Pennsylvania, Rhode Island, Vermont	New York Daily News
	New York Post
	Newsday
	Pittsburg Post-Gazette
	The Boston Globe
	The Buffalo News
	The Hartford Courant
	The Philadelphia Inquirer
	The Star-Ledger
**South**	
Alabama, Arkansas, Delaware, District of Columbia, Florida, Georgia, Kentucky, Louisiana, Maryland, Mississippi, North Carolina, Oklahoma, South Carolina, Tennessee, Texas, Virginia, West Virginia	Atlanta Journal-Constitution
	Orlando Sentinel
	San Antonio Express News
	Star-Telegram
	Sun Sentinel
	Tampa Bay Times
	The Arkansas Democrat-Gazette
	The Baltimore Sun
	The Courier-Journal
	The Dallas Morning News
	The Houston Chronicle
	The Oklahoman
	The Virginian Pilot
**West**	
Alaska, Arizona, California, Colorado, Hawaii, Idaho, Montana, Nevada, New Mexico, Oregon, Utah, Washington, Wyoming	Arizona Republic
	Honolulu Star Advertiser
	San Diego Union Tribune
	San Francisco Chronicle
	The Denver News
	The Orange County Register
	The Oregonian
	The Sacramento Bee
	The San Jose Mercury News
	The Seattle Times

* States and newspapers are listed in alphabetical order within their region; newspapers are not listed in association with the states.

**TABLE 2:** Characteristics of sexual violence articles in national and regional traditional media outlets, by region — United States, 2014–2017*

Characteristic	No. (%) of articles				
	Nationwide (n = 520)	Midwest (n = 520)	Northeast (n = 520)	South (n = 520)	West (n = 520)
**Type of sexual violence**					
Sexual assault	302 (58.1)^†^	322 (61.9)	341 (65.6)	345 (66.3)	320 (61.5)
Rape	242 (46.5)^§^	245 (47.1)^¶^	224 (43.1)	246 (47.3)**	194 (37.3)
Child sexual abuse	148 (28.5)^††,§§^	199 (38.3)	223 (42.9)^¶¶^	184 (35.4)	176 (33.8)
Sexual exploitation	233 (44.8)	243 (46.7)	223 (42.9)	239 (46.0)	263 (50.6)
Sex trafficking	41 (7.9)	52 (10.0)***	26 (5.0)^¶¶,†††^	59 (11.3)	63 (12.1)
Prostitution	33 (6.3)^§^	43 (8.3)	28 (5.4)^¶¶,†††^	55 (10.6)	61 (11.7)
Sexual harassment	144 (27.7)^§§§^	80 (15.4)	73 (14.0)	60 (11.5)**	100 (19.2)
Child pornography	19 (3.7)^††^	40 (7.7)	26 (5.0)	37 (7.1)	27 (5.2)
**Media language used**					
Sex scandal/Scandal	57 (11.0)^§§§^	25 (4.8)	28 (5.4)	31 (6.0)	18 (3.5)
Sex/Intercourse	89 (17.1)^§,††,§§^	56 (10.8)	44 (8.5)^†††^	83 (16.0)**	50 (9.6)
Accuser	69 (13.3)^§,††^	40 (7.7)	55 (10.6)	50 (9.6)	41 (7.9)
Accused	170 (32.7)^§^	134 (25.8)^¶¶¶^	144 (27.7)^†††^	186 (35.8)**	129 (24.8)
**Outcome/Prevention messaging**					
Consequences for perpetrator	180 (34.6)	208 (40.0)***	154 (29.6)	174 (33.5)	184 (35.4)
Call for secondary/tertiary prevention	117 (22.5)^§§^	118 (22.7)***	76 (14.6)	103 (19.8)	93 (17.9)
Call for primary prevention	65 (12.5)^†,§,§§^	50 (9.6)	31 (6.0)	31 (6.0)	38 (7.3)

* Comparisons are made between regions by type of sexual violence, media language used, and outcome/prevention messaging (p<0.05).

^†^ Nationwide significantly different from South.

^§^ Nationwide significantly different from West.

^¶^ Midwest significantly different from West.

** South significantly different from West.

^††^ Nationwide significantly different from Midwest.

^§§^ Nationwide significantly different from Northeast.

^¶¶^ Northeast significantly different from West.

*** Midwest significantly different from Northeast.

^†††^ Northeast significantly different from South.

^§§§^ Significantly different from all other regions.

^¶¶¶^ Midwest significantly different from South.

Coverage for the types of sexual violence was similar by year, except for significant differences in reporting during 2017 for rape, sexual exploitation, sex trafficking, and sexual harassment (Table 3). In 2017, reporting on rape and sex trafficking was significantly lower (34.9%, and 5.7%, respectively) than during 2014–2016 (46.8%–48.5% and 9.8%–10.9%, respectively; Table 3). Sexual harassment articles were more frequent in 2017 (35.7%) than in previous years (a low of 9.7% in 2014). Newspaper coverage in 2017 differed considerably from that in other years in media language used, with significantly more coverage than all other years for use of the term “sex scandal” or “scandal” (10.9%), “accuser” (15.4%), and “accused” (37.4%). In 2017, coverage of consequences for perpetrators (38.9%) was significantly higher than coverage in 2014 (31.5%). No significant differences by year regarding calls for primary, secondary, or tertiary prevention were found.

**TABLE 3:** Characteristics of sexual violence traditional media articles — United States, 2014–2017*

Characteristic	No. (%) of articles			
	2014 (n = 650)	2015 (n = 650)	2016 (n = 650)	2017 (n = 650)
**Type of sexual violence**				
Sexual assault	392 (60.3)	389 (59.8)	423 (65.1)	426 (65.5)
Rape	315 (48.5)	305 (46.9)	304 (46.8)	227 (34.9)^†^
Child sexual abuse	233 (35.8)	230 (35.4)	246 (37.8)	221 (34.0)
Sexual exploitation	251 (38.6)^§^	263 (40.5)	308 (47.4)	379 (58.3)^†^
Sex trafficking	71 (10.9)	69 (10.6)	64 (9.8)	37 (5.7)^†^
Prostitution	67 (10.3)^¶^	69 (10.6)**	53 (8.2)	31 (4.8)
Sexual harassment	63 (9.7)	78 (12.0)	84 (12.9)	232 (35.7)^†^
Child pornography	39 (6.0)	42 (6.5)	31 (4.8)	37 (5.7)
**Media language**				
Sex scandal/Scandal	25 (3.8)	24 (3.7)	39 (6.0)	71 (10.9)^†^
Sex/Intercourse	89 (13.7)	97 (14.9)**	75 (11.5)	61 (9.4)
Accuser	40 (6.2)^§^	47 (7.2)	68 (10.5)	100 (15.4)^†^
Accused	173 (26.6)	170 (26.2)	177 (27.2)	243 (37.4)^†^
**Outcome/Prevention messaging**				
Consequences for perpetrator	205 (31.5)^¶^	219 (33.7)	223 (34.3)	253 (38.9)
Call for secondary/tertiary prevention	134 (20.6)	143 (22.0)	107 (16.5)	123 (18.9)
Call for primary prevention	60 (9.2)	46 (7.1)	44 (6.8)	65 (10.0)

* Comparisons are made between years by type of sexual violence, media language used, and outcome/prevention messaging (p<0.05).

^†^ Significantly different from all other years.

^§^ 2014 significantly different from 2016.

^¶^ 2014 significantly different from 2017.

** 2015 significantly different from 2017.

## Discussion

Major differences in the type of sexual violence mentioned, media language used, and outcomes were identified by region, year, or both. Overall, a higher percentage of articles in national outlets than in regional outlets used sex scandal, sex/intercourse and included calls for prevention. In general, the type of sexual violence mentioned and the language used in 2017 differed from that during other years (e.g., decreased mention of rape and sex trafficking and increased mention of sexual harassment). These changes might reflect wider coverage of sexual harassment and exploitation allegations involving prominent figures in the film industry, media, state and national congresses, and technology companies, including the “#metoo” movement, which experienced a resurgence in the fall of 2017 that could have influenced article content during the last quarter of 2017.^§^

The findings in this report are subject to at least three limitations. First, research was limited by access to electronic databases that carried traditional print media newspapers; therefore, only 48 of the top 50 distributed newspapers in the United States were accessible. Second, although outlets were identified by reach and stratified by region, how much each publication outlet encompasses rural readership is unclear, and generalizations to these populations should be made with caution. However, many print outlets are also widely available online, likely increasing their reach beyond their physical distributions. Finally, this study did not examine how audiences interact with print and electronic news media through social media. For example, social media allows users to comment on and challenge how traditional news frames sexual violence (*10*). Such social media interactions present an opportunity for further research and consideration in understanding the complex impact of media on public perceptions of sexual violence.

Media reporting included both suggested language (e.g., “sexual assault”) and language to avoid (e.g., “sex scandal” or “scandal”), as referenced in the Sexual Violence Media Guide (*4*). Traditional media might have more of an impact on increasing awareness and prevention of sexual violence if their portrayals do not place blame on the victim and if they use suggested terms to describe violent acts throughout their articles. Focused dissemination of the Sexual Violence Media Guide (*4*) might benefit all media outlets.

Outcomes including perpetrator consequences or prevention messaging generally were reported infrequently. Although outcomes might not be known at the time of reporting, traditional media might be missing an opportunity to integrate prevention messages within current or breaking news. The media can play an important role by partnering with public health organizations to ensure that their portrayals of sexual violence are factual, nonbiased, do not inadvertently blame victims, and include prevention messages in stories about sexual violence. One of the prevention strategies identified in the STOP SV technical package, which includes the best available evidence to prevent sexual violence, is promoting social norms that protect against violence (*9*). As an institution that can influence social norms, the media might contribute to efforts to prevent sexual violence through accurate descriptions of prevalence and impact of sexual violence, establishment of sexual violence as a public health issue, and, when possible, inclusion of messages and resources for prevention. In this way, awareness of the problem and prevention messaging might reach broader audiences.

Understanding how media outlets have historically framed sexual violence might help public health officials and practitioners work productively with journalists to identify potential unintended effects of specific language use. The Sexual Violence Media Guide (*4*) can be used to inform and evaluate the impact of public health and media collaborations. The media, public health practitioners, and communities can work together to incorporate language from the Sexual Violence Media Guide (*4*) to change public perceptions about circumstances surrounding sexual violence and encourage public health approaches to prevention.

**Summary**

**What is already known about this topic?**

Sexual violence media portrayals can influence public perceptions, which can affect social norms and behavior.

**What is added by this report?**

Examination of articles from traditional print media outlets found regional and temporal differences in types of sexual violence covered, media language used, and outcomes reported in news story coverage in 2017, compared with that from 2014 to 2016.

**What are the implications for public health practice?**

Through cross-sectoral collaboration and use of the Sexual Violence Media Guide language suggestions, media, public health practitioners, and communities can work together to effectively use best practices to report on sexual violence, emphasize sexual violence as preventable, and frame sexual violence as a public health issue.

## Footnotes

* Boolean search for each publication: ((“sexual violence”) OR (“sexual assault”) OR (“sexual abuse”) OR (“child sexual abuse”) OR rape OR incest OR (“intimate partner violence”) OR (“sexual exploitation”) OR (“human trafficking”) OR (“sex trafficking”) OR prostitution OR (“sexual harassment”) OR exposure OR (“unwanted penetration”) OR (“unwanted sexual contact”) OR (“forced oral contact”) OR (“forced genital contact”) OR grope OR voyeurism OR (“alleged victim”) OR (“alleged perpetrator”) OR perpetrator OR (“sex scandal”) OR intercourse OR (“perform oral sex”) OR fondle OR accuser).

† Newspaper databases: News Bank Inc. (https://www.newsbank.com/); Gale OneFile (https://www.gale.com/databases/gale-onefile); US Newsstream (https://about.proquest.com/products-services/nationalsnews_shtml.html).

§ https://journals.sagepub.com/doi/10.1177/1940161220968081; https://metoomvmt.org.
